# Women’s acceptability of a self-collect HPV same-day screen-and-treat program in a high burden setting in the Pacific

**DOI:** 10.1186/s12913-022-08842-1

**Published:** 2022-12-12

**Authors:** Hawa Camara, Somu Nosi, Gloria Munnull, Steven G. Badman, John Bolgna, Joseph Kuk, Glen Mola, Rebecca Guy, Andrew J. Vallely, Angela Kelly-Hanku

**Affiliations:** 1 Kirby Institute for Infection and Immunity in Society, UNSW Sydney, Wallace Wurth Building, UNSW Sydney, Kensington, NSW 2052 Australia; 2 Papua New Guinea Institute of Medical Research, Homate Street, PO Box 60, Goroka, Eastern Highlands Province Papua New Guinea; 3 Department of Obstetrics and Gynaecology, Modilon General Hospital, PO Box 1200, Madang, Papua New Guinea; 4 Mt Hagen Provincial Hospital, PO Box 36, Mt Hagen, WHP 281 Papua New Guinea; 5 School of Medicine and Health Sciences, University of Papua New Guinea, PO Box 5623, Boroko, NCD Papua New Guinea

**Keywords:** Acceptability, Self-collect, Screen-and-treat, Cervical cancer, HPV testing, Early detection, GeneXpert, Papua New Guinea

## Abstract

**Background:**

A field trial to evaluate a self-collect point-of-care HPV screen-and-treat (HPV S&T) program was implemented in two Well Women Clinics in Papua New Guinea (Papua New Guinea). Assessing the acceptability of a health intervention is a core element of evaluation. In this study, we examined women’s acceptability of both self-collection and HPV S&T intervention in Papua New Guinea.

**Methods:**

Sixty-two semi-structured interviews were conducted with women who had undergone cervical screening in the same-day self-collected HPV screen-and-treat program in Madang and Western Highlands Provinces, Papua New Guinea. Data were thematically analysed using the Theoretical Framework of Acceptability (TFA) and managed using NVivo 12.5.

**Results:**

Most women agreed that self-collection was transformative: it helped circumvent the culturally embarrassing pelvic examination and increased their self-efficacy, especially due to the provision of health education, instructions, and pictorial aids. The availability of same-day results, and treatment if indicated, was particularly valued by the women because it reduced the financial and temporal burden to return to the clinic for results. It also meant they did not need to wait anxiously for long periods of time for their results. Women also appreciated the support from, and expertise of, health care workers throughout the process and spoke of trust in the HPV-DNA testing technology. Most women were willing to pay for the service to ensure its sustainability and timely scale-up throughout Papua New Guinea to support access for women in harder to reach areas.

**Conclusion:**

This study reported very high levels of acceptability from a field trial of self-collection and HPV same-day screen-and-treat. The program was deemed culturally congruent and time efficient. This innovative cervical screening modality could be the ‘solution’ needed to see wider and more immediate impact and improved outcomes for women in Papua New Guinea and other high-burden, low-resource settings.

**Supplementary Information:**

The online version contains supplementary material available at 10.1186/s12913-022-08842-1.

## Introduction

In November 2020, following recent advances in primary and secondary cervical cancer prevention, the World Health Organization (WHO) launched a new global strategy for the elimination of cervical cancer [1, 2]. Cervical cancer is the fourth most common cancer in people with a cervix (from here on, ‘women’) worldwide. Cervical cancer is particularly pressing in low-and-middle-income countries (LMIC) where the burden of disease is greatest, and access to preventive services have been beyond the reach of most. The new WHO strategy recommends that 70% of women be screened with a high-performance HPV test by age 35 years and again by age 45 years [2, 3].

Primary cervical screening using HPV-DNA testing is a high-performance test that has been shown to substantially out-perform the Papanicolaou (Pap) test and visual inspection with acetic acid (VIA) methods [4–8]. With portable closed molecular platforms able to detect HPV-DNA at or near point of care, HPV-DNA testing provides convenience, timeliness, and improved patient outcomes [9, 10], with test results provided the same day, reducing loss to follow up [10–12]. In settings where the risk of loss to follow up is high, WHO strongly supports a single-visit, same-day HPV screen-and-treat approach as part of its global strategy for elimination [2].

The introduction of self-collection (or self-sampling) for cervical screening allows women to collect their own vaginal specimen [13] and has comparable performance to clinician-collected sampling [7, 14–17]. Self-collection is less embarrassing and uncomfortable compared with a clinician-collected cervical swab and as such has the potential to increase the participation of never screened or under-screened women [18–21]. A recent qualitative meta-synthesis found that self-collection is widely accepted by women and a variety of other stakeholders due to three key factors: self-collection enhances self-efficacy, provides comfort, and ensures privacy [22, 23]. It also significantly increases service acceptability among women, providing social and systemic benefits, such as the reinforcement of peer support among women and easier access to the health system. New evidence from Australia corroborates this with similar findings, prompting a national move towards self-collection for all, not just under-screened, women [20, 21, 24].

This ‘same-day self-collect HPV screen-and-treat’ combination offers the potential for increased coverage and as a result more women can be provided timely treatment, reducing morbidity and mortality. As these technologies transform the cervical screening landscape, we need to ask ourselves, how acceptable are these technologies in the local settings where they are applied or are hoped to be applied in the future?

### Cervical screening in Papua New Guinea

Papua New Guinea (Papua New Guinea) is the most populous county in the Pacific and has a higher-than-average age-standardized (ASR) cervical cancer incidence rate of approximately 30 cases per 100,000 and a mortality rate of 20 cases per 100,000 women, ahead of all countries in the Oceania region [178]. Data from 2019 show that cervical cancer is the second most common cancer among women aged 15–44 years of age, with an estimated 1024 new cases per year (25.9 annual crude incidence rate per 100,000 in 2018), and accounts for 663 deaths per year [25] and 28% of cancer hospital admissions [26].

Earlier approaches to cervical screening, including Pap test-based and VIA methods, were suboptimal due to a variety of implementation issues, notably the former method’s long results turnaround time and significant loss to follow up, as well as issues of clinical performance with the latter. Building on earlier research led by members of our team [27, 28], a large-scale two-site prospective intervention trial among women in Papua New Guinea was conducted to evaluate a novel, fully integrated strategy comprising point-of-care HPV-DNA testing of self-collected vaginal specimens followed by same-day thermal ablation for women in need of treatment (HPV S&T) [6, 7]. In addition to confirming the clinical performance of this strategy, other studies aimed to assess the cost-effectiveness [29] and health systems requirements of the approach, as well as the acceptability of the strategy for women and other stakeholders.

### Theoretical approach to acceptability

Acceptability, the extent to which an intervention, approach or program is agreeable to a group of people [30], is a key component for those engaged in implementation research. In previous studies there have been inconsistencies in the applicability and definitions of acceptability. While quantitative studies seek to numerate acceptability, by providing the proportion of participants who are accepting of an intervention, for example, qualitative research seeks to understand in depth and through narratives the extent to which diverse aspects of an intervention are acceptable in relation to attitudes, socio-cultural factors, quality of care, preferences, and patient-provider interaction [31–38]. In response to ongoing ambiguity in defining acceptability, Sekhon and colleagues [39] undertook a systematic approach to identify key constructs instrumental in assessing the acceptability of health interventions. The Theoretical Framework of Acceptability, or TFA, defines acceptability as ‘a multi-faceted construct that reflects the extent to which people delivering or receiving a healthcare intervention consider it to be appropriate, based on responses to the intervention’ (p. 4). Sekhon and colleagues also identified seven key constructs for the TFA, as shown in Table 1 [39, 40].

**Table 1:** The Theoretical Framework of Acceptability and its seven component constructs

Component Construct	Definition
Affective Attitude	How an individual feels about the intervention
Burden	The perceived amount of effort that is required to participate in the intervention
Ethicality	The extent to which the intervention has a good fit with an individual’s value system
Intervention Coherence	The extent to which the participant understands the intervention and how it works
Opportunity Costs	The extent to which benefits, profits or values must be given up to engage in the intervention
Perceived effectiveness	The extent to which the intervention is perceived as likely to achieve its purpose
Self-efficacy	The participant’s confidence that they can perform the behaviour(s) required to participate in the intervention

When used in qualitative research, the TFA can be helpful in several ways. Before the implementation phase, the framework can be applied to help design acceptable health care interventions. During the trial phase, it can serve to modify/improve the intervention. And to ensure scalability, the framework can help assess the acceptability of all key stakeholders [39].

As new screening technologies are implemented as part of global efforts to eliminate cervical cancer, it is important to understand the experience of women to whom the technology is being introduced. Most of the previous qualitative literature focuses on traditional cervical screening methods (i.e., Pap smears, VIA). There are few qualitative studies focusing on women’s experience of self-collection [41] and none that examine self-collection *and* same-day screen and treatment. This is the first study of its kind using a qualitative methodology to elicit women’s perceptions of a same-day self-collected HPV screen-and-treat program.

We applied the TFA to examine women’s acceptability of the HPV S&T intervention in Papua New Guinea and consider the wider implications of our findings for future implementation and scale-up of this strategy in Papua New Guinea and in other high-burden, low-resource settings.

## Methods

The data drawn on for this paper are part of a qualitative study nestled within a field trial of a self-collect same-day HPV-DNA screen-and-treat program.

### HPV-based self-collect, test, and treat screening strategy: setting, eligibility, and procedures

**Fig.1:**
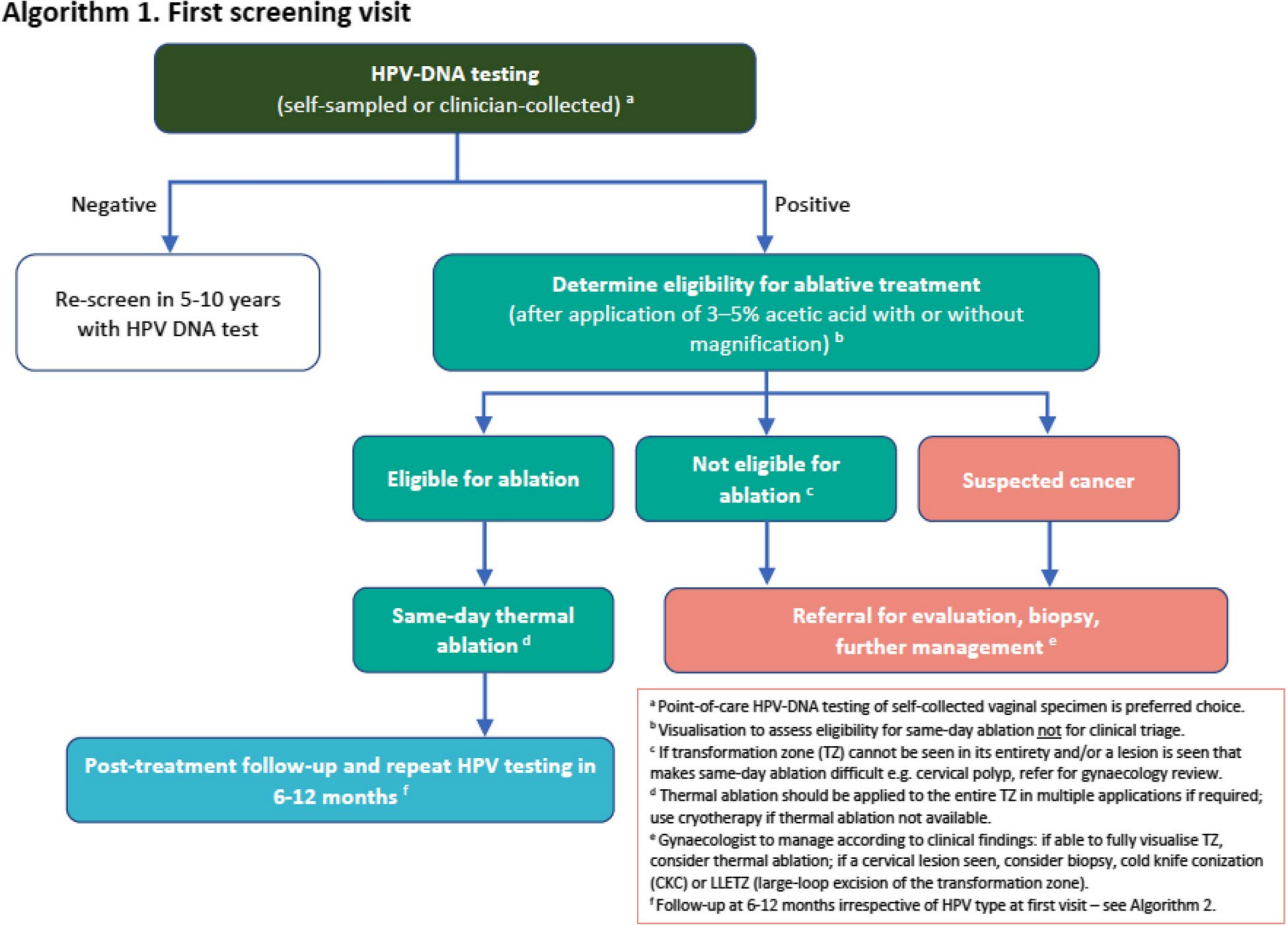
HPV S&T Clinical Algorithm

The field trial study was conducted at two Well Women Clinics (WWC) in Papua New Guinea: Modilon General Hospital, Madang Province and Mount Hagen General Hospital, Western Highlands Province. These sites were decided upon by national health experts in Papua New Guinea as well as provincial health authorities, and other local stakeholders. The key criteria that were used to assess the suitability of clinic sites included the presence of an existing dedicated Well Woman Clinic (WWC) and suitable clinic space available for the establishment of HPV screen-and-treat to cater for > 200 women per month as well as an experienced consultant obstetrician/gynaecologist to treat advanced cases of disease identified through the program. A robust pre-intervention training program was established where health care workers (HCW) were employed and trained to provide same-day HPV screen-and-treat including a comprehensive health education, operation of the closed molecular platform used (GeneXpert), application of thermal ablation for oncogenic/high-risk HPV (or hr-HPV) positive women, and post-test counselling. See Fig. 1 for the HPV S&T clinical algorithm used.

Prior to self-collection the WWC nurses routinely provided a 10–15-min group health education session (‘tok save’ in Papua New Guinea Pidgin) to all women in attendance at the clinic, in the morning before their appointment. The WWC’s health care workers led the session using a script provided by senior investigators of the trial. The session provided information on the signs and symptoms of cervical cancer, its association to HPV, and detailed instructions on how to perform self-collection. Examples of unused self-collection kits were also shared with women, allowing them to feel the specimen collection device (a soft conical-shaped cytobrush) and to ask questions before self-collection. The services were provided free of charge to all women.

### Study sample and recruitment

As a qualitative sub-study, we conducted a total of 62 semi-structured interviews with women who had attended the WWC and participated in HPV S&T program from May 2018 to August 2019. Women who met the eligibility criteria for the clinical trial and had undergone same-day self-collection, testing (and, if needed, treatment), were eligible to participle in this qualitative study. Purposive sampling was used to ensure a heterogeneous sample by identifying women who would provide insightful, diverse, and information-rich narratives addressing the research question at hand, including women who had tested positive for hr-HPV and required thermal ablation [42].

The women interviewed were approached by the WWC staff after their first visit and invited to participate in the interview.

Interview guides were designed to understand women’s overall experience with the HPV S&T program, as well as each element of the program, such as the health education talks, their understanding of cervical cancer and HPV, self-collection, testing, and, for the women who had tested positive for HPV, treatment. Questions were also asked about program sustainability, including willingness to pay for future services.

### Data collection and analysis

Interviews were conducted in Papua New Guinea Pidgin (Tok Pisin) or English or a mix of both and were audio-recorded, transcribed, and translated prior to analysis. The interviews were conducted by the first and second authors, the second author being an experienced female Papua New Guinean social researcher fluent in both English and Tok Pisin.

Before the interview, all participants were given an information and consent form in a language of their choice (Tok Pisin or English). The form was explained to each of the participants before starting the interview to ensure all participants understood the purpose of the interview, provided consent to participate, and signed the consent form before starting. The interviews took place at convenient locations, ensuring the participants’ (and researchers’) audio privacy and security. The participants were provided with light refreshments for their time. All participant identifiable information was deidentified and all names used in this paper are pseudonyms.

An inductive-deductive thematic analysis [43, 44] was used to analyse the data using NVivo 12.5 software [45]. After familiarisation with the data, the first author iteratively conducted a line-by-line coding to identify patterns. From these patterns, a codebook was developed. Codes and quotes were identified and categorized into themes. The first author subsequently utilized a deductive approach using the TFA’s seven component constructs as the coding frame to map the themes found in the inductive analysis phase. The first author assessed and assigned each of the themes against the TFA component constructs. The final thematic structure, as outlined in the findings section, was reviewed, and agreed upon with all co-authors.

### Ethics

This study was approved by the Papua New Guinea Institute of Medical Research Institutional Review Board (IRB) (No. 1712), the Medical Research Advisory Committee (MRAC) of the Papua New Guinea National Department of Health (NDoH) (No. 17.36), and the Human Research Ethics Committee at UNSW Sydney (No. HC17631). All methods were carried out in accordance with relevant guidelines and regulations.

## Findings

### Sample characteristics

The median age of the women interviewed was 41 years and the majority reported being married (*n* = 36, 58%) and employed (*n* = 40, 65%). Most women had been formally educated and almost all were affiliated with a Christian church. See Table 2 for more demographic details.

**Table 2:** Demographic data of female participants

Demographics		Madang		Mount Hagen		Combined	
		*n* = **36**	%	*n* = **26**	%	*n* = **62**	%
**Age range**	30–39	15	42	12	46	27	44
	40–49	10	28	10	38	20	32
	50–49	6	17	3	12	9	15
	N/A	5	14	1	4	6	10
**Marital Status**	Single	3	8	0	0	3	5
	In a relationship/ Married	19	53	21	81	40	65
	Separated/ Widow	10	28	5	19	15	24
	N/A	4	11	0	0	4	6
**Number of children**	0	5	14	1	4	6	10
	1—2	11	31	8	31	19	31
	3 +	15	42	15	58	30	48
	N/A	5	14	2	8	7	11
**Employment Status**	Yes	29	80	11	42	40	65
	No	2	6	4	15	6	10
	N/A	5	14	11	42	16	26
**Education**	Bachelor	11	31	1	4	12	19
	Diploma/ Certificate	10	28	8	31	18	29
	Up to Grade 12	9	25	14	54	23	37
	No school	0	0	3	12	3	5
	Postgraduate	2	6	0	0	2	3
	N/A	4	11	0	0	4	6
**Religion**	Baptist	1	3	1	4	2	3
	Catholic	9	25	5	19	14	23
	Evangelical	2	6	3	12	5	8
	Lutheran	9	25	2	8	11	18
	Pentecostal	5	14	8	31	13	21
	Seventh Day Adventist	1	3	2	8	3	5
	United Church	2	6	0	0	2	3
	N/A	7	19	5	19	12	19
**Province of Origin**	Madang	15	42	0	0	15	24
	Western Highlands	0	0	21	81	21	34
	Other	21	58	5	19	26	42

All women approached during recruitment agreed to participate in the qualitative interviews. The interviews were on average 40 min long; some were as brief as 25 min and others were an hour and a half long.

The findings section highlights each of the TFA constructs (see Table 1) while detailing specific sub-themes identified during the inductive analysis process. Illustrative quotes are provided in Appendix A.

### Affective attitude

The construct of Affective Attitude details women’s feelings about participating in HPV S&T. This section examines the following areas of affective attitudes: (1) feelings about self-collection, (2) views of health care workers, (3) benefits of getting tested, and (4) experience of being treated with thermal ablation.

#### Feelings about self-collection

From an acceptability point of view there are two aspects to assessing ‘self-collection’. First is the procedure of self-collection itself and the second relates to the experience of the brush collection device used in the procedure.

Concerning the procedure, most agreed that being able to collect their own vaginal sample was acceptable, with both comfort and privacy as main reasons. Albeit initially intimidated by being asked, shown, and tasked with taking their own sample, most women appreciated the procedure.

When women attended the WWC for ‘check-ups’, the HCWs provided the women with a pictorial aid of how to perform the self-collection procedure. Once the self-collection demonstration was completed, women would individually go to the private space allocated (i.e., toilets) in the clinic to collect their specimen. Most of the women expressed that the private nature of the sampling method increased their willingness to attend and comfort in being screened.

Of the women interviewed, 24 reported that they had previously had a Pap smear; they emphasized their frightening and invasive pelvic examination experience. In contrast, these women deemed self-collection as painless, accommodating of their desire for privacy, and easy to use. Comfort and ease were closely related to the material of the brush, not only the process, describing it as comfortable and painless. While a small group of women were initially afraid, women were surprised at and welcomed the softness of the brush, alleviating their fears.

#### Views of health care workers

The HCWs’ attitudes towards the women were highly influential in the participants’ overall experience, and therefore acceptability, of HPV S&T. The majority of the women expressed that the HCWs were understanding, knowledgeable, and giving of their time.

This patient-provider relationship was central to why participants reported sharing their experiences with other women in their communities, resulting in a considerable increase of women presenting at the WWCs. For many of the women, the patient-provider interaction at the clinic was in stark contrast to their past experiences with HCWS.

#### Benefits of screening

For all women, undergoing screening was an active way to protect and maintain their health and prevent premature death from a disease they assessed as being widespread in their communities.

Even women who tested positive for hr-HPV and required further treatment expressed relief in knowing that they could do something to look after themselves and that their participation in the service meant that they had received lifesaving treatment.

#### Experience of being treated with thermal ablation

After receiving their results, the women who tested positive for hr-HPV were treated on the same day with thermal ablation. The majority of women felt relief after treatment and believed they had been cured.

A few of the women who tested positive and needed subsequent treatment shared the discomfort of feeling the heat being applied to their cervix, and consequently concerned about the possibility of adverse effects. Very few experienced any pain and or discomfort post-treatment.

After receiving treatment, health care workers advised the women that they were to abstain from sexual intercourse for six weeks. Most of the women who were in a relationship experienced support from their male partner; disclosing their treatment experience to their partner/husband led to them receiving support and empathy from their significant other. There was, however, fear from some that the need to abstain from sex for six weeks may cause other health-related issues.

### Burden

Closely related to perceived effectiveness and self-efficacy, the construct of burden highlights the perceived amount of effort required to participate in the intervention.

The majority of the women perceived the self-collection process as easy and requiring minimum effort on their part. Women who preferred clinician collected samples were the exception.

### Ethicality

This component of the TFA centres on the extent to which a program is perceived to be a good fit with the participants’ value system. For increased acceptability, it is imperative to assess whether this program would align with the women’s cultures and values.

Most women spoke of the intervention addressing major cultural barriers which have hindered women from participating in previous screening programs. The intervention, as it was re-designed with self-collection and same-day screen-and-treat strategy, was now culturally congruent and significantly increased women’s attendance at the participating clinics. Most communities in Papua New Guinea perceive sexual and reproductive health matters as taboo. One key deterrent to Papua New Guinean women’s participation in screening programs is the pelvic examination. Women have avoided participating in previous cervical screening programs due to the shame and embarrassment associated with it. A major feature of the intervention is the use of self-collection to circumvent the pelvic examination.

Another main feature was referenced that addresses local needs. The program, led by female health care workers, ensured women’s comfort, and facilitated their engagement in the service in what they described as a ‘safe’ environment. The availability of the self-collection option will increase uptake of young women and women who have not yet given birth in cervical cancer screening programs.

### Intervention coherence

Intervention coherence relates to the extent to which participants understand the intervention. In this section, we will explore the impact of health education and instructions provided by the health care workers.

Most women acknowledged the impact of clear instructions and algorithms. The availability of visual aids in the private space (i.e., toilet door) reserved for the self-collection procedure benefitted the women while they were collecting their specimen and did not require high levels of literacy.

Confirming the previously discussed *views of health care workers* sub-theme, women appreciated health care workers’ willingness to answer any of their questions. Most women noted that the health care workers were thorough and as detailed as possible.

### Opportunity costs

This construct expands on the extent to which benefits, profits or values must be given up to engage in the intervention. In this section, we review the sub-themes of (1) same-day screen-and-treat and time efficiency, (2) cost of service, and (3) willingness to pay.

#### Same-day screen-and-treat and time efficiency

For many of the women, same-day screen-and-treat was viewed as time efficient, a significant feature of this program. That translated into reduced travel time, and less time off work and away from family duties. It also ensured that eligible women were promptly treated (i.e., reducing loss to follow-up).

#### Cost of service

All women were enthusiastic about having access to a free screening service. The free-of-charge service increased accessibility to all women, especially those who resided in remote villages and/or were unemployed. The financial advantage that HPV S&T brought was compared to previous cervical screening programs, The HPV S&T program could potentially address this barrier.

#### Willingness to pay

All of the women acknowledged the benefit of the screening service and expressed that they would still attend if the program became fee-for-service in the future. They truly believed that the benefit of the screening program and knowing their status outweigh the cost of the service. The women noted that it was preferrable for the service to remain free of charge due to the potential widespread lack of finance. Despite this, the women provided estimates that they believed would be a reasonable charge for the screening service should there be a need. The recommended cost of the service varied between 5 and 200 Kina (PGK), equivalent to USD1.40 to USD57, noting that women in remote and rural areas and/or unemployed, thus at higher risk for cervical cancer due to lack of access, should be eligible to pay a lower fee, while women in more stable conditions and/or with insurance could afford the higher cost range:

Other considerations included the women’s location of residence: depending on where they resided, they would have to pay for transportation costs, increasing the total cost required to attend the service.

### Perceived effectiveness

This construct assesses the extent to which the program is perceived as likely to achieve its purpose. This section will elaborate on the women’s views of the closed molecular platform used to test for HPV and raising awareness.

#### Views of ‘the machine’

The introduction of the testing platform, referred to as ‘the machine’ by most women, was found to be one of the key factors in women’s acceptability of the program. Most women saw the value in the testing technology and trusted the results.

Most women acknowledged that further examination by the HCW to confirm their test results was deemed unnecessary because they trusted the machine. The ‘machine’s’ time-sensitive feature was seen as an additional benefit to treat women on time.

#### Raising awareness

Beyond their own individual benefits, some women saw how their participation in the wider program could lead to greater benefits for the country; early detection and treatment would reduce the burden of the disease in Papua New Guinea. Women who have gone through the screening, regardless of their diagnosis, decided to share their experience to raise awareness. Most women expressed that they were undergoing the intervention so that they could openly share their experience in the hopes of encouraging others to attend, due to cervical cancer being so pervasive in their communities. The women raising awareness resulted in significant uptake, highlighting the high acceptability of the current trial as designed and the unmet need for more accessible screening programs in the country. Most women recognized the need for community awareness on HPV and its link to cervical cancer and, with the changing dynamics and demographics (i.e., early teenage pregnancy rates on the rise), young girls also face high risks of contracting STIs, such as the oncogenic HPV strains.

### Self-Efficacy

Self-efficacy is defined as participants’ confidence that they can perform the behaviour required to participate in the program. For this study, self-efficacy relates to the self-collection procedure.

Self-efficacy plays an immense role in the acceptability and uptake of self-collected HPV screen-and-treat. Having a major role in your care, especially reproductive health care, is considered unprecedented and potentially empowering, but can also be unnerving for some. The women in the study fell at either end of the spectrum: lacking or having self-efficacy.

The majority of women felt confident about performing self-collection. Closely aligned with the *impact of health education and instructions* sub-theme (under intervention coherence), all the women enthusiastically agreed that the health education sessions provided them with the confidence they needed to perform self-collection and to accept treatment.

A few women preferred clinician-collected screening because they didn’t trust their abilities to perform the test correctly or thought it could also be an opportunity for women to cheat their test results.

## Discussion

Most of the qualitative research conducted to date has focused on women’s experiences of the Papanicolaou (Pap) smear and/or VIA programs [46]. This is the first qualitative study to explore women’s acceptability of a HPV S&T program in Papua New Guinea, and to our knowledge, globally, using the TFA. The use of the TFA has allowed to examine women’s experiences from key angles, including key constructs such as affective attitude and self-efficacy. The findings have shown the interwoven complexities of the personal, cultural, and contextual dimensions of women’s acceptability of a point of care HPV-based same-day screen-and-treat intervention in Papua New Guinea. Our findings indicate that women’s acceptability of our screening strategy was influenced by several inter-related aspects. The women articulated numerous factors, particularly, the opportunity to collect their own vaginal samples, feeling welcomed by trusted health care workers, the testing and treatment conducted on the same day, and the (free) cost of the service.

The *affective attitude* component demonstrated the importance of feelings towards an intervention. This component construct was a combination of different key elements. A trustworthy and empathetic health care worker is a crucial element of the cervical screening experience and considerably influenced women’s acceptability of the program. Previous research substantiates this finding; studies from Switzerland and Australia have shown how the patient-provider relationship heavily impacts women’s acceptability of similar programs, and self-collection in particular [47, 48]. The most salient element was the self-collection procedure. In low-resource settings, where the majority of women have never undergone screening for cervical cancer, the use of self-collection can be intimidating [13]. Nevertheless, the privacy and comfort it provides made it easily acceptable to women. Self-collection is less invasive, allows for confidentiality and privacy, is convenient and practical, and removes embarrassment from the pelvic examination. These findings are in line with previous research highlighting the key attractive features of the collection method, including the brush’s characteristics [23, 41, 49–54]. It is also seen as an opportunity to learn about and become more comfortable with one’s body [47, 49, 55] while practising self-care [56].

As described in the self-efficacy construct, the women developed a sense of *empowerment*. Previous research has emphasized women’s perceived ability to perform self-collection, highlighting their autonomy and control of their bodies [47, 49, 54, 55]. The data also show the contrast between having or lacking self-efficacy. Previous studies highlighted this internal conflict between *trust in self* to perform the self-collection procedure or whether to have the health care worker perform the procedure. As highlighted in previous research, some women preferred clinician-collected screening because they lack confidence in their abilities to perform the test correctly [49, 57–60]. Further research could explore key factors that increase and/or reduce women’s self-efficacy to perform self-collection, as well as women’s preferences of the method of collection.

Health education was influential in the intervention coherence of the program. Health care workers provided clear instructions to perform self-collection, as well as an opportunity to address women’s questions and fears about cervical cancer. Previous research has highlighted similar findings that feelings such as embarrassment and fear could be alleviated with proper knowledge and education about the screening process [51, 55, 61, 62]. Women are also more trusting of the results when they are provided with instructions before performing the self-collection procedure, relating it closely to the perceived effectiveness of the program [63]. Further research could explore the design and types of health education aids that could influence the acceptability of the intervention, considering literacy levels and cultural realities.

Under the ethicality component, the theme of cultural congruence was highly significant. Cultural norms have an immense impact on individuals’ perspectives of health care services and of their local realities in developing countries. Previous studies conducted in the country have shown the importance of cultural norms on women’s health care practices [64–70]. This study’s findings emphasized how the program’s design fits within the country’s cultural norms. Self-collection and same-day screen-and-treat address some of the major cultural and structural barriers that hinder women’s participation in cervical screening programs. In addition, albeit self-collection was accepted by women of all ages, a few participants over 45 years of age stated that self-collection could be used to increase uptake of younger women. Research has shown that women of childbearing age will be more accepting of self-collection [58].

Lastly, the sub-themes under the opportunity costs construct showcase key factors that women are willing to forego to access the service. Cost [47, 55, 63] and time [54, 71, 72] are key elements, consistent with previous research highlighting the need to address barriers to cervical screening acceptability. A recent study has demonstrated the program’s cost-effectiveness [29]. Yet behavioural economic studies should be a focus for further research to better understand the impact of the actual cost of service to women, their willingness to pay, and how local governments can help financially support these services in the future.

Strengths of this study include the relatively large sample size for qualitative research, including women from each of the study sites, as well as heterogeneity of the women, ensuring that our analysis can speak to a wide variety of experiences. The qualitative interviews allowed us to critically examine all components of the TFA.

While the framework provided a strong foundation for our analysis, this qualitative study was not initially designed with the TFA in mind. It would have been useful to include it in the initial stages of the study design for a more comprehensive interview guide leading to richer data.

## Conclusion

This qualitative study explored the experience and acceptability of a same-day point-of-care self-collect HPV screen-and-treat program for cervical screening and treatment among women in a high-burden low-and middle-income country setting. Key features included the use of self-collection and its device, the empathy of health care workers, and the same-day availability of molecular HPV test results and acceptable treatment options. This study shows that this innovative screen-and-treat modality is widely accepted and could potentially increase screening uptake, resulting in wider impact and improved outcomes for women in Papua New Guinea and other high-burden, low-resource settings. 

## Supplementary Information

**Additional file 1:** **Appendix A.** Illustrative quotes.

## Data Availability

The data used and analyzed for this study were not made publicly available to protect participants’ privacy but are available from the corresponding author on reasonable request.
