# Supplementary material for: Women’s acceptability of a self-collect HPV same-day screen-and-treat program in a high burden setting in the Pacific
Source: BMC Health Serv Res. 2022 Dec 12;22:1514. doi: 10.1186/s12913-022-08842-1 (PMC9746197; doi:10.1186/s12913-022-08842-1)
Supplement: Supplementary file 1 — Additional file 1: Appendix A. Illustrative quotes. [file 12913_2022_8842_MOESM1_ESM.docx]

**Appendix A. Illustrative quotes**

| Component Construct | Theme | Quotes |
| --- | --- | --- |
| Affective Attitude | ***Feelings about self-collection*** | The health care workers won’t see you… you aren’t exposed; it wasn’t painful, it was comfortable.  (Berecia, 48 years old)  This special brush won’t hurt or affect our vagina whatsoever.  (Ayla, 42 years old).  I felt bad, I didn’t feel good about going up on the bed and opening my legs because you know I feel shy. But with that [self-collection] the check-up is so easy and so efficient for us.  (Ayla, 42 years old) |
|  | ***Views of Health care workers*** | When I went inside, I was ashamed but when the nurses smiled at me greeted me, I felt at ease and happy because sometimes when we are sick and go there [to the hospital], just the tone of their voices make our sickness grow bigger. I saw that they listened to patients and that they are good people.  (Dina, 35 years old)  Those are the kind of health worker we want and not those, you know, hard face looking ones, no smile. Just imagine, you trying to get some information and it’s hard and it’s not even nice. But these three ladies [HPV S&T health care workers] they are number one.  (Serafina, 52 years old)  At some other clinics, they [health care workers] are so stubborn and ignorant, like if you feel like you want to ask them any questions, just by looking at them, you will be like ‘uh… it is ok, forget it, I will just ask someone else’, because they are not friendly… The health care workers here were very friendly, and I liked it.  (Daria, 32-year-old) |
|  | ***Benefits of Screening*** | I came here today because you know I have to be alive to work and I even came on Wednesday. I left my class and came today as well because I have to be alive to keep on doing this you know, so life is very important so my coming here did not whatever [affect] other things. It is good.  (Diana, 33 years old)  The results come out that same time and you know your status. You know where you are and so you can help yourself.  (Rina, 52-years old). |
|  | ***Experience of being treated with thermal ablation*** | The most important thing is that I have been treated. And I know that the treatment will help me get cured. I was having discharges, slight bleeding and then pus and I felt like it’s a bit sore… the cervix. But after treatment, it [all] went away.  (Belina, 39 years old)  It did some changes in me. Inside, it was really hot, and she [the health care worker] said if you feel like it’s very hot, you tell me, and I will stop. So we stopped, and we did it again. She applied the heat again. It was painful inside.  (Mera, 30-year-old)  That time I was treated, I was feeling good when they heated me up. But three days later, I feel this pain, ‘how did this thing happen?’ Maybe it was done in wrong way or that’s what I was thinking. So, I went to the doctor, and I said, ‘please see what is going on’ because this pain is under my belly button.  (Amy, 34 years old)  If we refuse sex with our husbands, he could go out [have an extra-marital affair] and get himself infected with HIV and then, bring that virus into the family.  (Maeira, 33 years old)  Because she just treated me with heat, I might damage myself when trying to clean myself or when inserting my fingers inside trying to clean myself or something like that. That was what I was thinking. I figured out that heat has been applied and my inside is probably soft so in the process of cleaning, I might hurt myself.  (Serafina, 52 years old) |
| Burden | ***N/A*** | I’d just prefer something like this ‘cause it’s much easier. Like get the sample our self, and that’s it; we just wait for an hour and get our results.  (Daria, 32 years old)  This is similar to the toothbrush, similar way [we are] brushing our teeth, insert into the vagina and get the specimen to be tested. It’s easy.  (Dina, a 35-year-old)  I didn’t like it very much… I just want to lay comfortably on the bed and you people [health care workers] do the test on me.  (Rina, 52 years old) |
| Ethicality | ***N/A*** | That technique is very good because different people [can participate]. [Initially] they will think, ‘will I be examined by a male or female?’ This [self-collected brush] gives them the opportunities to help them[selves] so they can come forward and really take part in this so that if they have a problem then, you know, they can be cured.  Maybe young ladies will come too. Like, we mothers will not be ashamed, but you see for young ladies, it will be like this [feel ashamed]. Now, they will feel comfortable.  (Brenda, 40 years old) |
| Intervention Coherence | ***N/A*** | I asked few questions about the different stages because she actually showed us what the different [stages of] cervical cancer [looks] like or how does it look like, explained how heat was applied, what changes will take place and all this, so she was pointing at it and was explaining it. It will look like this and after that, it will look like this. It was very educational.  (Mera, 30 years old)  They [health care workers] explained properly and demonstrated it [self-collection], I followed the instructions carefully and even in the toilet, there was the instruction with pictures showing how to insert [the brush] and so that was helpful too’  (Elsa, 43 years old). |
| Opportunity Costs | ***Same-day screen-and-treat and time efficiency*** | I knew my results, and I will come back and follow-up… which is good. Not like before, you will be tested and will wait some time for the results and in regard to the distance, transportation, money power, which is very difficult but now it is ok, that is why I am encouraging mothers to come forward.  (Belina, 39 years old) |
|  | ***Cost of service*** | The opportunity was there but the problem was the money. For Pap smear checks when you have the money, you go you pay, they check you. But now, you are giving this opportunity free to all kinds of women.  (Maya, 40 years old).  If it was a private clinic for me, I think I would spend so much money to get this kind of treatment but with what you guys are doing here and giving us free of charge for that, ah it’s a bonus.  (Bernita, 40 years old). |
|  | ***Willingness to pay*** | Fifty kina because I am talking on behalf of those who are not employed, PGK50 is a standard amount that everyone can come up with to pay. If it’s PGK100, we working mothers can afford, but what about those who are not working?  (Jenny, 42 years old)  Twenty kina… I am thinking about the bulk of the population. Most of them are unemployed and they are not residing here where the clinic is. They have to travel a distance where it is costly. That is why the fee should be at least a minimum that they can afford.  (Rose, 37 years old) |
| Perceived Effectiveness | ***Views of the machine*** | Yes, I trust the machine. It’s like something new. And it’s good that it’s here. I was just tested, I knew my results and I’m gone, and I will come back and follow-up.  (Jenny, 42 years old)  I went inside with a doubt, when the sister [HCW] said your result is negative, an unexplainable kind of joy got me. She did not see my inside, but I felt very grateful.  (Diana, 33 years old).  The machine reads quickly if you already have that virus in there and it can prepare the mothers like, the early stage so the staff [can] explain to them what is going on.’  (Briana, 48 years old) |
|  | ***Raising awareness*** | I want more awareness to go right down to the women because as it is now, when you talk about such things, a lot of women still think that it’s a disease that belongs to women who are currently living with men. [They believe that] this disease is not for women that don’t have [a] man. But what about those women that don’t have men but have died from this disease? And it’s these kinds of people who are getting sick.  (Sayra, 46 years old)  It’s good for them to know their status because nowadays cancer cases are happening almost like Malaria… not like before… like HIV too… so I told them that I will give awareness and I’m doing that in my area. I am encouraging women to come for [the] test.  (Lina, 32 years old).  It’s serious because babies are having babies, how can a baby look after a baby? This awareness has to go right down to the schools.  (Sayra, 46 years old). |
| Self-efficacy | ***N/A*** | It felt so good by just following the instructions, she [the health care worker] did a demonstration like when you are cleaning your ears with cotton buds in your ear. So, I got that picture, and I did that in my cervical whatever.  (Ayla, 42 years old).  Because it's me, I'm going to help myself to get the collection instead of the doctors or some other medical things [provider] check me.  (Carrel, 36 years old)  It felt good, I felt that I could do that. I don’t want someone else to do it for me, I can do it myself. Sometimes it’s uncomfortable having someone else do it for me.  Marla, 32 years old)  After I did the test myself, I went back and told [the health care worker] ‘I don’t think I did it well. I don’t like myself to do it, I want you to do it’. Yeah, because I feel that if we do it ourselves, we will not do it properly.  (Imani, 42 years)  I prefer the doctor. Sometimes we might not insert the brush. We might cheat... I think we might cheat, yes. So, it's good for the doctors to help us, so they would find the disease in us.  (Carole, 39 years old) |
